# Supplementary material for: Chromothripsis during telomere crisis is independent of NHEJ, and consistent with a replicative origin
Source: Genome Res. 2019 May;29(5):737–49. doi: 10.1101/gr.240705.118 (PMC6499312; doi:10.1101/gr.240705.118)
Supplement: Supplemental Material [file supp_gr.240705.118_Supplemental_file_1.zip › contigs/annotated_contigs/DB79/contig.2.DB79_length_727_mean_cov_7.04264099037.docx]

**DB79_length_727_mean_cov_7.04264099037**

```
GAGCTGAGTGAATGCCTGGGTCTGGTGGCTTTCTCCACCACCACTGCCGAGTCCCGTACGTCCTGTGCAACACAGGGAAGAGGAAGGGT
 >chr9:138300543-138300947 + E=3e-228
GATAGTTCACAGGCACCAGCACTTTTACAGGTGTTCATTCATTACACCATTCTCCATCTGAGGAAACTGGGACCCAGCGAGGTTGAGCA

ACGTAGTCAAAGTCACACAGCTGATAAGTCATAGAACTGATATTCAAGGCCCAGCTCTGCAGCCCCAGACATTGTGCTATGCCACCTAA

ACCAGCCAGAAGAACATCCTGGGAAGGGTGCAAAGGAAGCTGGCGGTGGGCCAGCAGGTGTGAAGGGGCCCAGGGCTGGGAGGAAGATG

AAGACACAAAGTGACCCGAGTGGGGCTGCTGCCCCGGGGCCCTCTGCC|ATTCTTGGAGA|AACGACTGGACTGGTTTTCTCCCCTTTC
                                                            >chr9:114293122-114293434 +
CTAGATCCAGTCTCTCAAGATTCTACTGTTTCCCAAGAGGAGAACTCAAAAGAGGATAAAATGGTCACTGTTTGTCCCAATACTGAGTC
 E=7e-170
CTGTGTAAGTTTCCTTTGATGGTTTTTATTCCTAAGTGAATACTTAATTGAGCTCCTACTGAGTGGTATAAAGATATAAAAATAAAAGG

CTAGGCACGGTGGCTTACACCTGTAATCGCAGCACTTTGGGAGGCTGAGGCAGGAGGATCGCTTGAGCCCAAGAGTTCAGGGTTGCAGT

GAGCTATGATCATGCCA
```
